# Image Watermarking Using Least Significant Bit and Canny Edge Detection

**DOI:** 10.3390/s23031210

**Published:** 2023-01-20

**Authors:** Zaid Bin Faheem, Abid Ishaq, Furqan Rustam, Isabel de la Torre Díez, Daniel Gavilanes, Manuel Masias Vergara, Imran Ashraf

**Affiliations:** 1 Department of Computer Science & Information Technology, The Islamia University of Bahawalpur, Bahawalpur 63100, Pakistan; 2 School of Computer Science, University College Dublin, D04 V1W8 Dublin, Ireland; 3 Department of Signal Theory and Communications and Telematic Engineering, University of Valladolid, Paseo de Belén 15, 47011 Valladolid, Spain; 4 Center for Nutrition & Health, Universidad Europea del Atlántico, Isabel Torres 21, 39011 Santander, Spain; 5 Universidad Internacional Iberoamericana, Arecibo, PR 00613, USA; 6 Universidade Internacional do Cuanza, Cuito EN250, Angola; 7 Área de Nutrición y Salud, Universidad Internacional Iberoamericana, Campeche 24560, Mexico; 8 Fundación Universitaria Internacional de Colombia, Bogotá 111311, Colombia; 9 Department of Information and Communication Engineering, Yeungnam University, Gyeongsan 38541, Republic of Korea

**Keywords:** least significant bit, substitution box, image watermarking, cryptography

## Abstract

With the advancement in information technology, digital data stealing and duplication have become easier. Over a trillion bytes of data are generated and shared on social media through the internet in a single day, and the authenticity of digital data is currently a major problem. Cryptography and image watermarking are domains that provide multiple security services, such as authenticity, integrity, and privacy. In this paper, a digital image watermarking technique is proposed that employs the least significant bit (LSB) and canny edge detection method. The proposed method provides better security services and it is computationally less expensive, which is the demand of today’s world. The major contribution of this method is to find suitable places for watermarking embedding and provides additional watermark security by scrambling the watermark image. A digital image is divided into non-overlapping blocks, and the gradient is calculated for each block. Then convolution masks are applied to find the gradient direction and magnitude, and non-maximum suppression is applied. Finally, LSB is used to embed the watermark in the hysteresis step. Furthermore, additional security is provided by scrambling the watermark signal using our chaotic substitution box. The proposed technique is more secure because of LSB’s high payload and watermark embedding feature after a canny edge detection filter. The canny edge gradient direction and magnitude find how many bits will be embedded. To test the performance of the proposed technique, several image processing, and geometrical attacks are performed. The proposed method shows high robustness to image processing and geometrical attacks.

## 1. Introduction

Information Technology (IT) has revolutionized the world with major advancements in every field of life. Its applications in different domains such as image processing [1], edge computing [2], computer vision [3,4,5], health care [6], internet of things (IoT) [7] and many more are helping to build a smart and digital world. Digital technology is so widespread that our personal lives depend on these technologies. Nowadays, a person feels lonely without digital technology. Moreover, people use online banking, shopping, trades, marketing, health care, education, and much more. The internet has made this world a single virtual unit and made communication easier and faster. With the use of the internet and digital technology, every field makes advancements and solves its future issues with these technologies. Companies move from physical resources to online resources to maintain their budgets, continuity, and energy. These technologies are evolving so fast that they impact the confidentiality of individual and company information. Due to advancements in communication technology, the security of digital data has become a major challenge today [8].

Cryptography is a security domain that provides security services, i.e., integrity, authenticity, and confidentiality [9]. In cryptography, data are encrypted before moving to an insecure channel, and a key is sent to decode that data. Digital data security is most important nowadays because people rely on digital technology. To fulfill the trust of people, educational and business firms give more attention to digital data security [10]. Cryptographic algorithms from the cryptosystem are used to secure communication between two parties. Cryptosystems can be symmetric and asymmetric cryptosystems where the symmetric cryptosystems have the same key, while asymmetric cryptosystems have different keys for encryption and decryption. The strength of the cryptosystem depends on the substitution box [11].

Substitution Box (S-Box) is a nonlinear component in the cryptosystem that produces dispersion in the generated sequence. Due to its major role, researchers try to improve the design of S-Box. In this research, the watermark image is scrambled by our chaotic S-Box. S-Box with high nonlinearity and low differential probability is cryptographically strong and improves the strength of the cryptosystem [12,13]. S-Box creates dispersion and prevents the attacker from suggesting input to the output. S-Box can be categorized into algebraic S-Box, chaotic S-Box, and heuristic S-Box. In this research, an improved chaos-based S-Box with strong cryptographic properties is used. Chaos is a domain of nonlinearity that shows nonlinear behavior with lower computation. Due to fewer computations and high nonlinearity, researchers have explored chaotic S-Boxes from the last decade [14,15]. S-Box is used in data hiding techniques to scramble the hidden data to improve security.

Image watermarking is a data-hiding technique used for copyright protection and content authentication. Watermarking is a technique to hide a watermark signal in a carrier signal to prove the authenticity of the carrier signal [16,17]. Watermarking can be categorized into text, image, and video watermarking. In text watermarking, a watermark is implanted into the text carrier signal, while in image and video watermarking, a watermark is embedded into the image and video carrier signal. Watermarking can be implemented into two broader domains, i.e., spatial domain and frequency domain. The spatial domain is simple, and manipulation is directly on the pixel level and has a high payload capacity. The least significant bit (LSB) embedding is an example of a spatial domain. While the frequency domain is complex and works on a frequency component [18]. Discrete cosine transform and discrete wavelet transform are examples of the frequency domain. Watermarking is mostly divided into two phases: the first one is the embedding phase, while the second one is the extraction phase. In the embedding phase, the watermark is implanted into the carrier signal, while in the extraction phase, the watermark is recovered from the watermarked image or video. In addition, watermarking can be visible or invisible if considered visually. In visible watermarking, the watermark is seen by the naked eye, and it is used for content authentication, while in invisible watermarking, the watermark cannot be seen by the naked eye and is used for copyright protection. Based on adversarial attacks, watermarking can be robust watermarking and fragile watermarking. In robust watermarking, the watermark resists adversarial attack and cannot be destroyed, while in fragile watermarking, the watermark is destroyed after slight modification [19]. With the innovation of information technology and multimedia, the threat of digital content misuse always remains under consideration. Therefore, certain effective measures are needed to protect digital content.

Several works on image watermarking have been presented. Rishi et al. [20] proposed a machine learning-based image watermarking approach in which the Mersenne Twister generator is used to generate a random number, and based on the random number, the positions for watermark embedding are selected. The proposed technique shows robustness against different attacks, but imperceptibility values are not comparable. Due to the low imperceptibility value, the watermark is prominent and easy to detect. The proposed technique is good but lacks in some areas, i.e., imperceptibility. Daming Li et al. [21] proposed a Convolutional Neural Network (CNN)-based image watermarking technique using Discrete Cosine Transform (DCT). The proposed technique gives promising results with the cost of computational overhead. The proposed technique lacks computational efficiency. Wafa et al. [21] proposed an image watermarking scheme based on single value decomposition (SVD) and integer wavelet transform to improve robustness, security, and imperceptibility. The proposed scheme generates a hash value to overcome the false positive problem. Hasan et al. [22] used encryption-based image watermarking in two-level discrete wavelet transform and discrete cosine transform. The proposed scheme shows high robustness against different image processing attacks, but geometrical attacks are not covered by the proposed approach. Moreover, the proposed approach shows peak signal-to-noise ratio (PSNR) values greater than forty and structural similarity index measure (SSIM) values closer to one. Yang Liu et al. [23] proposed an encryption-based image watermarking technique in the spatial and frequency domains. The watermark image is first scrambled and then embedded in the host image. The PSNR value of the proposed technique is good and provides robustness against multiple attacks. The recovered watermark against some attacks is not very good, but the overall technique is good and gives good results. Savakar et al. [24] combined blind and non-blind techniques to achieve more robustness and imperceptibility. The proposed technique gives good results in terms of PSNR values. The major issue with this approach is complexity. First, blind watermarking is performed then non-blind watermarking is performed, which makes it computationally more complex. Durgesh et al. [25] solved the false positive problem of SVD by adding the discrete cosine coefficient of each least significant and most significant bit of gray image into a singular middle value. Salah Mokhnache et al. [26] proposed an image watermarking approach using a gradient imaging filter and discrete wavelet transform (DWT). The gradient is used to find the appropriate places for watermark embedding. The proposed technique gives better results but in most cases, the extracted watermark is damaged. The comparison between existing studies is shown in Table 1.

Shuangming Yang et al. [32] proposed an improved spiking neural network (SNN), which is helpful in dealing with the recent challenges of machine learning-based systems. The proposed technique improves the performance of SNN with a minimum error entropy technique. The performance of the proposed approach is tested on memory and autonomous navigation analysis. The SNN uses a plausible neuron model on dynamic rate, while the existing neuron-based algorithm ANN uses a static rate. Machine learning approaches use the learning process to perform a cognitive task, while continual meta-learning provides next-level machine intelligence. Similarly, ref. [33] proposed spike-Driven Few-Shot Online Learning with entropy theory to deal with recent challenges in the learning process. The state-of-the-art SNN is not robust in learning, while the entropy-based SNN enhances the learning process. The existing deep neural network consumes high power compared to SNN. The learning capability of the machine learning algorithms and SNN are limited and dependent on label data, while entropy theory-based SNN enhances the learning level and accomplishes the physically impossible task with minimum resources.

Technology facilitates different domains of life, but at the same time, it also drives us to an insecure zone. In the field of information technology, the invention of internet technology has also introduced data insecurity due to a large data movement on the internet. The digital data moves freely on insecure channels, and attackers can hack and modify the data. The digital data owner who wants to make revenue from his product always remains in danger because of the data breach. There is a need to make certain effective measures to secure digital content on a global level. In this paper, an image watermarking technique based on LSB and a canny edge detection algorithm are proposed. In this approach, an image is divided into non-overlapping blocks, and the intensity gradient of each block is found. Then, a convolution mask is applied to each block to find the gradient magnitude and direction. Based on magnitude and direction, the non-maximum suppression is applied, and the edges in that direction are found. The same process is repeated on each side based on gradient magnitude and direction. Finally, the watermark is added on the edges’ direction. If a watermark is added on smooth image areas, then the watermark can be easily detected, which pauses and breaks the security of the digital content. The main aim is to provide robustness and imperceptibility. The proposed approach uses LSB, which is computationally less expensive and provides a high payload. Secondly, in the proposed approach, the watermark signal is first scrambled and then added to the carrier signal, which increases the security of the proposed approach. This approach gives good results in terms of robustness and imperceptibility.

The remaining paper is structured as follows. Section 2 describes the preliminaries related to the current research work. Section 3 describes the materials and methods used in this research. Section 4 discusses the results of the proposed approach and comparison with existing works. In the end, Section 5 provides the conclusion of our research work.

## 2. Preliminaries

### 2.1. Image Gradient

**Figure 1:**
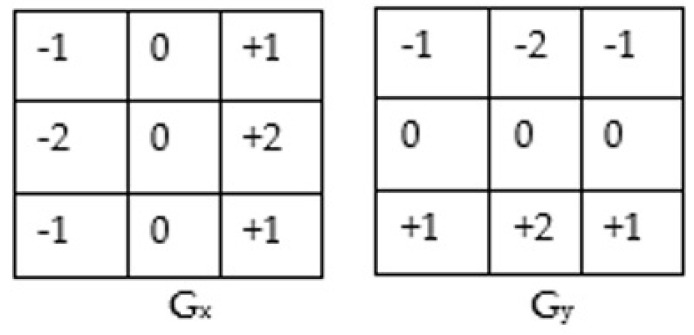
Convolution mask for canny edge detection.

The convolution mask is used to find the gradient magnitude and direction. The gradient magnitude provides information on the image variation. It tells where image sharpness occurs, while gradient direction provides the direction of image variation where image sharpness exists [34]. These two parameters help in image watermarking and provide information about positions feasible for watermark insertion and extraction. The convolution masks used for canny edge detection are [35]. Figure 1 shows the Convolution mask for canny edge detection.

The formula for magnitude and direction finding is given below.

(1) $$M=\sqrt{g_{y}^{2}+g_{x}^{2}}$$

(2) $$\lambda =tan^{-1}\left(\frac{g_{y}}{g_{x}}\right)$$

where *M* shows the magnitude and λ shows the direction, while $g_{y}$ indicates the *y*-direction gradient and $g_{x}$ indicates the *x*-direction gradient.

### 2.2. Least Significant Bit

**Figure 2:**
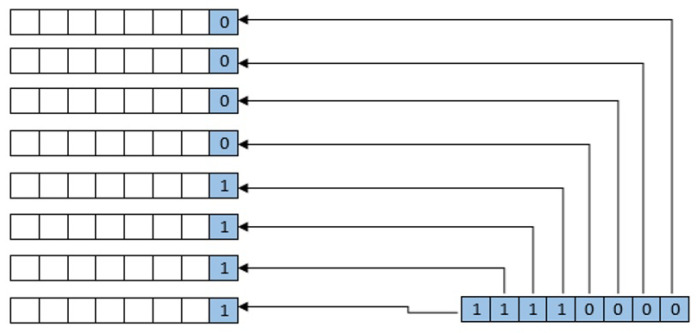
Embedding with message byte.

LSB is a simple spatial domain watermark embedding approach. In this approach, the watermark bit is inserted at the rightmost side of the pixel. LSB is a simple but robust technique in nature due to its direct pixel-level manipulation [36,37]. The mechanism to embed a watermark is shown in Figure 2.

### 2.3. Chaotic Substitution Box

The substitution box is a major element in the cryptosystem, and a cryptosystem’s strength depends on the substitution box. The substitution box basically generates dispersion in the generated sequence following Shannon’s principal [38]. S-Box is an auxiliary table that takes the nonlinear relation from input to output. S-Box in the early period was algebraic and heuristic; algebraic S-Boxes are computationally complex as a lot of mathematical operations are involved in generating S-Box. Chaos-based S-Box is computationally less expensive because chaos is a nonlinear system that is highly sensitive to the initial condition. By slightly changing the initial value, a completely new S-Box can be generated [39]. In this paper, a chaos-based S-Box based on a piecewise linear chaotic map (PWLCM) and optimization technique is used. The reason for choosing this S-Box is that PWLCM has a constant density function and is very effective in application [40]. PWLCM is represented by the following mathematical expression

(3) $$x_{n+1}=\left\{\begin{array}{cc} \frac{x_{n}}{\rho }, & 0\leq s_{n}<\rho \\ \frac{\left(x_{n}-\rho \right)}{\left(0.5-\rho \right)}, & \rho \leq x_{n}<0.5\\ \frac{\left(1-\rho -x_{n}\right)}{\left(0.5-\rho \right)}, & 0.5<x_{n}<1-\rho \\ \frac{\left(1-x_{n}\right)}{\rho }, & 1-\rho <x_{n}<1.0 \end{array}\right.$$

$X_{0}\in \left[0,1\right)$ and ρ is the control factor ρ∈(0,0.5).

The pseudo-code for the generated S-Box is given in Algorithm 1. By changing the value of $x_{n}$ and ρ, new S-Box values are generated. This is because chaotic S-Boxes are highly sensitive to an initial condition. The optimization technique chooses the values that map more difference between output values.

The S-Box values generated by the above algorithm are shown in Table 2. These S-Box values are used to scramble the watermark signal to add extra security.
**Algorithm 1** S-Box generation.**Input:** $X_{n},\rho ,I$**Output:** S-Box   1:  **while** (*i* < 300) **do**  2:     iterate PWLCM with $x_{n}$  3:     set $x_{n+1}=x_{n}$  4:     X⟵Floor(x×256)  5:     **if** X∉S−Box **then**  6:         Sub-Box ⟵X  7:         = i=i+1  8:     **else**  9:         iterate PWLCM with $x_{n}$10:     **end if**11:     Optimization12: **end while**

## 3. Materials and Methods

Watermarking is a field of data hiding, in which data is hidden in multimedia content in such a way that no one knows the presence of the hidden data. The hidden data maintains the property of imperceptibility, robustness, and security. The imperceptibility property shows that the original image and the watermarked image are the same. If the watermarked image is different from the original image, then the attacker easily knows that it is a watermarked image. Secondly, the robustness property ensures that the attacker’s attack does not break the inserted watermark. In this paper, an image watermarking technique based on LSB and canny edge detection is proposed. The LSB technique is used because it is simple and has high payload capacity, which means that more watermark bits are inserted. The more watermark bits are inserted, the more robust the technique will be. Moreover, the canny edge detection technique is used to find the suitable positions where watermark bits will be inserted. In this approach, first the gradient magnitude and direction are found. Later, based on that, the edges are found, and the watermark is inserted. The proposed technique is more secure because of its insertion pattern and scrambled watermark image. By this technique, the PSNR and normalized correlation (NC) values make a major difference compared to state-of-the-art methods.

### 3.1. Watermark Embedding

**Figure 3:**
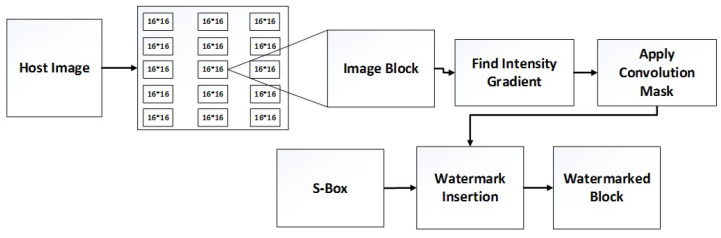
Watermark embedding flow diagram.

The image watermarking process comprises two main steps: watermark embedding and watermark extraction. Watermark embedding shows the sequence of steps to insert a watermark into the carrier signal. Figure 3 shows the flow diagram of image watermark embedding.

The watermark embedding process comprises the following steps:

**Step 1:** Original image *G* is divided into 16 × 16 independent blocks

(4) $$G=\{G_{1}\left(16\times 16\right),G_{2}\left(16\times 16\right),\ldots ,G_{N}\left(16\times 16\right)\}$$

**Step 2:** The intensity gradient of each block is found.

**Step 3:** The convolution mask is applied, and the gradient magnitude and direction of each block are found.

**Step 4:** Central and feasible pixels are selected for watermark insertion and separated into LSB and MSB.

(5) $$G_{N}\left(LSB,MSB\right)=G_{N}\left(X_{1},Y_{1}\right),\;N=\left\{1,2,3,\ldots ,256\right\}$$

**Step 5:** Addition of watermark bits using Equations (1) and (2).

  **Case 1:** $M\geq max(\frac{M}{2})$ Less bits are embedded into the LSB.

  **Case 2:** $M<max(\frac{M}{2})$ More bits are embedded into the LSB.

**Step 6:** The watermark image is scrambled using the chaotic S-Box.

**Step 7:** The watermark image is constructed by combining LSB and MSB.

**Step 8:** Considering all the above-mentioned steps, they are reverse performed.

### 3.2. Watermark Extraction

**Figure 4:**
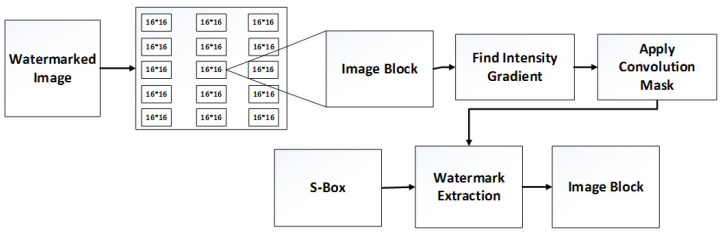
Watermark extraction flow diagram.

Watermark extraction is a process of extracting an inserted watermark from the watermarked image. The normalized correlation parameter shows whether the watermark is successfully extracted or not. The watermark extraction process is performed by reversing all steps, as mentioned in Section 3.1. Figure 4 shows the flow diagram of image watermark extraction.

## 4. Results and Discussion

To prove the effectiveness of the proposed approach, multiple image processing and geometrical attacks are performed. The most important parameters to test the proposed approach are PSNR, NC, and SSIM. The proposed approach is implemented on the Windows operating system, Intel Core i5 processor with 8Gb random access memory (RAM), and 6th generation Dell machine. MATLAB software is used to perform the operations. The SIPI image dataset is taken from the University of Southern California for experiments, which is available via http://sipi.usc.edu (accessed on 1 September 2022). The experiments involve the use of the grayscale image of size 512 × 512 and the watermark image of size 32 × 32. The watermark image is the first scramble by S-Box based on PWLCM. The LSB method is used to embed the watermark into the host image. The watermarked image is tested against different image processing and geometrical attacks.

### 4.1. Perceptual Quality Measure

To observe the physical appearance of the watermarked image, two important parameters must be calculated, i.e., PSNR and SSIM. The PSNR value measures the visual appearance. The large value of PSNR shows the visual equivalence between the original and watermarked image. On the other hand, SSIM measures the pixel similarity between the original and watermarked image. In image watermarking, both original and watermarked images look similar; this is the characteristic of the watermarking technique.

(6) $$\mu_{x}=\frac{1}{T}\sum_{i=1}^{T}x_{i},\;\frac{1}{T}\sum_{i=1}^{T}y_{i},$$

(7) $$\sigma_{x}^{2}=\frac{1}{T-1}\sum_{i=1}^{T}\left(x_{i}-{\bar{x}}^{2}\right),\;\sigma_{y}^{2}=\frac{1}{T-1}\sum_{i=1}^{T}\left(y_{i}-{\bar{y}}^{2}\right),$$

(8) $$\sigma_{xy}^{2}=\frac{1}{T-1}\sum_{i=1}^{T}\left(x_{i}-\bar{x}\right)\left(y_{i}-\bar{y}\right)$$

The average values of *x* and *y* are $\mu_{x}$ and $\mu_{y}$, respectively. The values of variance of *x* and *y* are $\sigma_{x}^{2}$ and $\sigma_{y}^{2}$, respectively. The covariance value of *x* and *y* is $\sigma_{xy}$.

The mathematical calculations for SSIM are given as

(9) $$SSIM\left(x,y\right)=\frac{\left(2\mu_{x}\mu_{y}+c_{1}\right)\left(2\sigma_{xy}+c_{2}\right)}{\left(\mu_{x}^{2}+\mu_{y}^{2}+c_{1}\right)\left(\sigma_{x}^{2}+\sigma_{y}^{2}+c_{2}\right)}$$

The watermark invisibility can be calculated by PSNR value as

(10) $$PSNR=10long_{10}\left(\frac{{\left(255\right)}^{2}}{MSE}\right)$$

where the 255 value is the maximum pixel value in the grayscale image, while MSE is the mean squared error, which is calculated as

(11) $$MSE=\frac{1}{M\times N}\sum_{m=1}^{M}\sum_{n=1}^{N}e{\left(m,n\right)}^{2}$$

**Figure 5:**
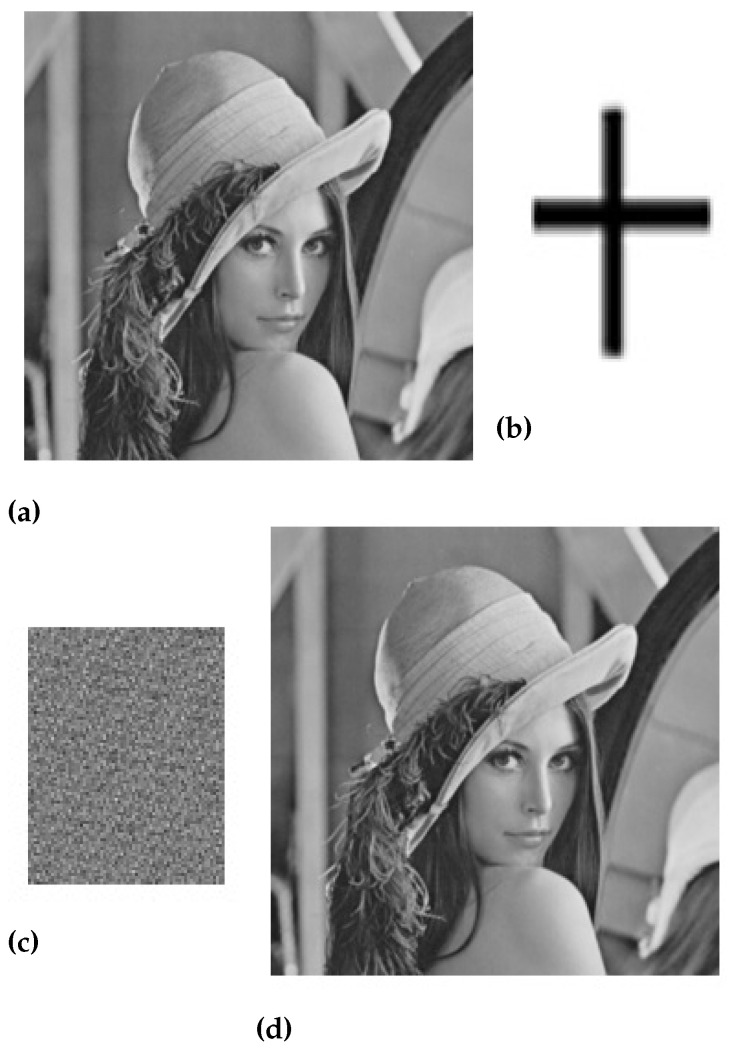
(**a**) Original Lena image, (**b**) Watermark image, (**c**) Scrambled image, and (**d**) Watermarked Lena image.

**Figure 6:**
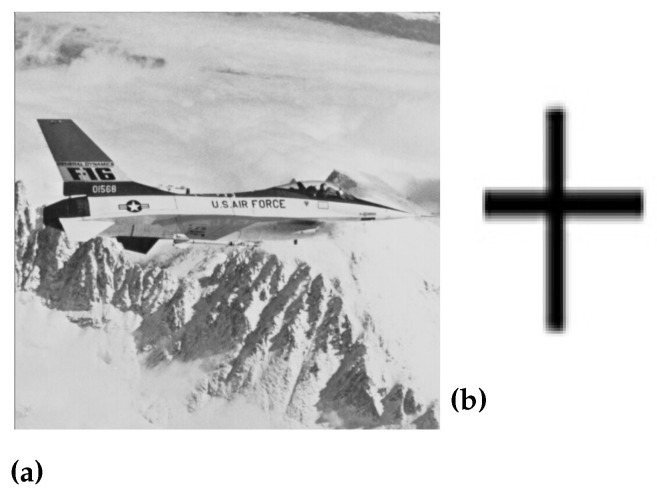

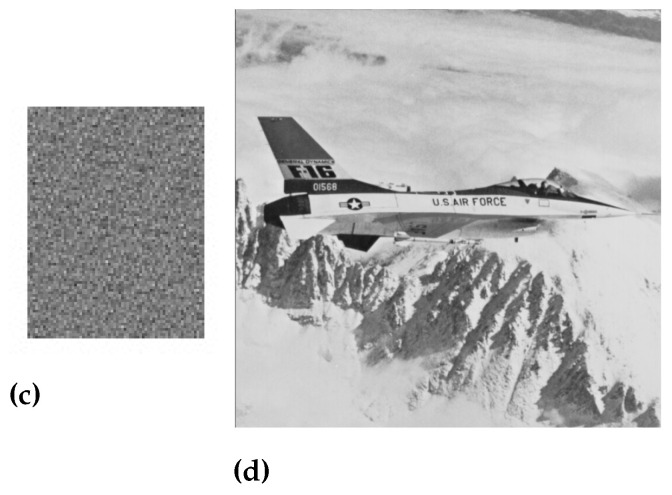
(**a**) Original jet image, (**b**) Watermark image, (**c**) Scrambled image, and (**d**) Watermarked jet image.

**Figure 7:**
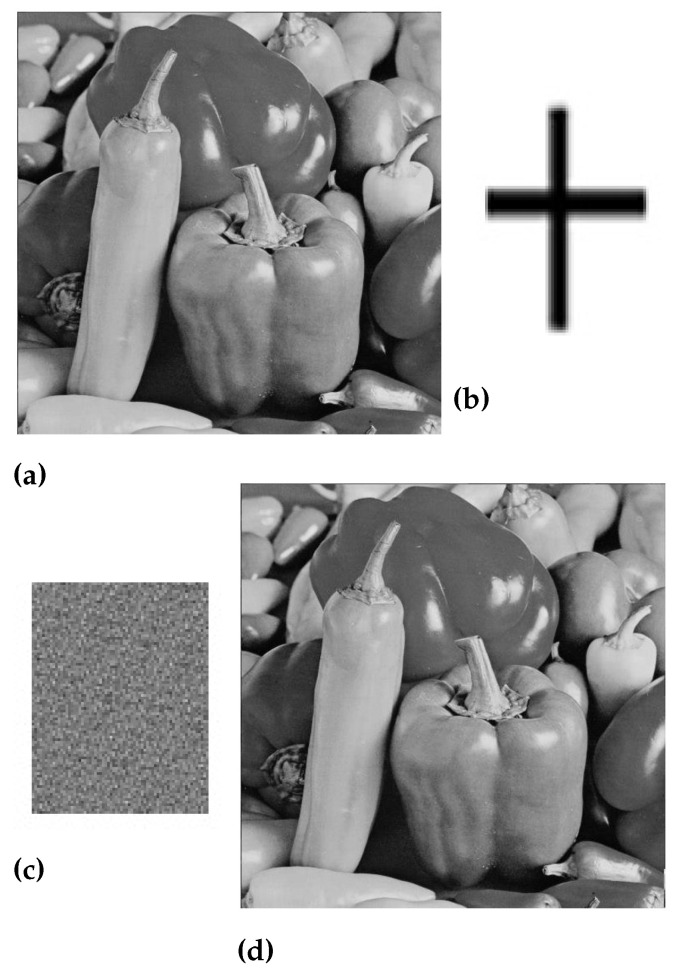
(**a**) Original peppers image, (**b**) Watermark image, (**c**) Scrambled image, and (**d**) Watermarked peppers image.

**Figure 8:**
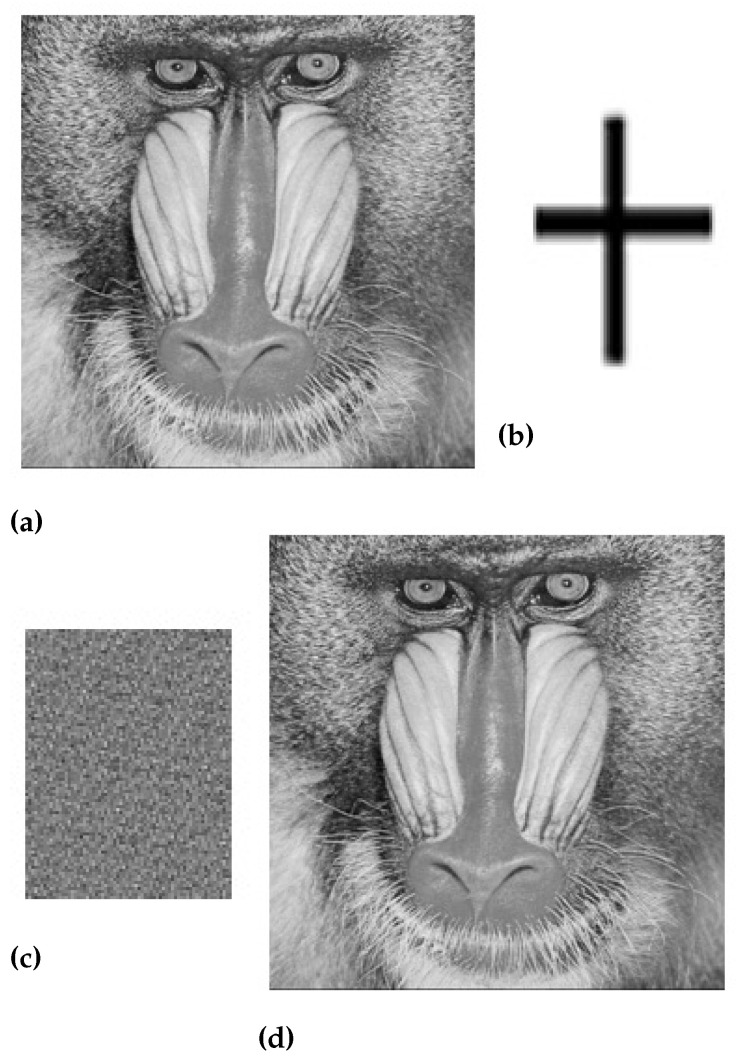
(**a**) Original baboon image, (**b**) Watermark image, (**c**) Scrambled image, and (**d**) Watermarked baboon image.

In the above equation, $e{\left(m,n\right)}^{2}$ is the measure of the difference between two images while M×N is the actual size of the original image. Figure 5, Figure 6, Figure 7 and Figure 8 are showing the impact of the watermarked approach on a sample of images.

The value of the perceptual quality measure parameter for the standard Lena and Baboon watermarked images without any attack is shown in Table 3.

### 4.2. Watermark Robustness

The resistance provided by the watermarking technique during the outbreak is termed robustness. Normalized correlation is the parameter to check the robustness of the watermarking technique. For effective watermarking, the normalized correlation value should be closer to or equal to one. If the normalized correlation is closer to one, it indicates that the watermark signal is safely extracted and no attack affects the watermarked image. This proves that the image is safe and not tampered with. The mathematical expression to calculate the normalized correlation is

(12) $$NC=\frac{\sum_{i}W_{ij}\sum_{j}w_{ij}^{\prime }}{\sum_{i}\sum_{j}{\left(w_{ij}\right)}^{2}}$$

where the original watermark value at (i,j) is $W_{ij}$, while extracted watermark value at (i,j) is $W_{ij}^{\prime }$.

The robustness analysis of the proposed technique against different image processing and geometrical attacks is shown in Table 4. These results are calculated on baboon images, which are taken from a standard SIPI image data set. The proposed technique based on the canny edge detection algorithm shows good results against image processing attacks, i.e., salt and pepper attacks, and Gaussian attacks.

The robustness of the proposed technique against geometrical attacks is shown in Table 5. The geometrical attacks concern the geometry of the image. This type of attack directly affects image dimensions and breaks the watermark signal. The aim is to save the watermark signal and safely recover the signal to prove its authenticity.

### 4.3. Comparison with Other Techniques

In this section, the performance of the proposed approach is compared with other image watermarking techniques. The comparison made with these papers because of current research is in a particular field. The comparative techniques cover the impact of modern tools and techniques, simplified, and cover the unexplored gap. One of the major benefits of the proposed technique is the smaller number of mathematical operations involved in this approach. Study [25] proposed a technique based on discrete cosine transform (DCT), DWT, and SVD. This technique is free from the false positive problem and provides promising results. The PSNR and NC of this approach are compared in Table 6. The promising result of the proposed technique is compared with [25,26] and [34].

Now attacks are performed on the proposed approach, and the results are compared with other approaches. The comparison of the proposed technique results with [26] shows the robustness of the proposed technique against image processing attacks, as shown in Table 7.

Table 8 shows the robustness against image processing attacks on Lena’s image. The comparison of the proposed technique results with [25] shows the robustness of the proposed technique against image processing attacks.

Table 9 shows the robustness against image processing attacks on baboon images. The comparison of the proposed technique results with [34] shows the robustness of the proposed technique against image processing attacks.

Now, geometric attacks are performed on the proposed technique, and the performance is compared with other techniques. Geometric attacks change the structure of the image and easily destroy the embedded watermark. Therefore, techniques are required to successfully extract the embedded watermark. Table 10 shows the robustness of the proposed technique against geometric attacks.

### 4.4. Discussion

This study provides a simple yet efficient method to perform image watermarking for security. The proposed approach is based on the canny edge detection algorithm and LSB approach. For watermarking, suitable places are found using canny edge detection. For performance appraisal, experiments are performed using different image processing and geometrical attacks, which shows that the proposed approach is robust against different types of attacks. Compared with existing approaches, the proposed approach provides better results in terms of SSIM and PSNR.

## 5. Conclusions

In this paper, an image watermarking technique based on a canny edge detection algorithm and LSB approach is proposed to embed watermarks. The canny edge detection algorithm is used to find suitable places to embed watermarks. It is a simple yet efficient approach to detecting edges in an image to find places more suitable to embed the watermark because on a smooth surface, attackers easily detect and break the watermark. Moreover, LSB is used to embed watermarks because LSB works on the pixel level in the time domain and has a higher capacity to add watermarks. In order to check the robustness of the proposed approach, different image processing and geometrical attacks are performed. The robustness parameter’s normalized correlation gives a value closer to one, indicating that the watermark is highly robust and did not break. To vary imperceptibility, SSIM and PSNR give good values that show that no visual change in the original and watermarked image is found. Experimental results indicate better results compared to existing state-of-the-art works. In the future, we intend to apply a new filter to measure the robustness of the watermark image. It is important to manage the filter type and watermark recovery quality. Furthermore, we want to implement this technique in color image watermarking and video watermarking.

## Figures and Tables

**Table 1:** Analytical comparison of existing works.

Technique	Image Type	Benefits	Limitations	Applications
[27]	Color	Robust against image processing attacks	Mathematically complex	Digital data security
[28]	Color	High robustness and high visual quality	Difficulty in image difference	Patent protection
[29]	Grayscale	Easiness in watermark extraction	Fragile	Content authentication
[30]	Grayscale	Robust against structural attacks. Removes false positive	Semi robust against histogram attacks	Content authentication
[31]	Grayscale	Robust against geometrical attacks	Mathematically complex	Digital circuits

**Table 2:** Generated substitution box.

	1	2	3	4	5	6	7	8	9	10	11	12	13	14	15	16
1	179	182	224	91	159	102	75	150	151	215	234	211	145	212	85	17
2	105	95	197	147	183	181	117	188	187	255	137	9	26	41	32	83
3	189	161	52	171	93	22	223	193	236	2	228	62	227	1	172	162
4	74	166	33	126	155	140	201	63	119	149	133	191	10	25	254	167
5	99	142	207	252	176	218	40	97	232	64	199	86	131	160	190	158
6	173	23	20	19	113	129	251	165	198	70	15	237	244	128	139	61
7	122	5	130	121	214	21	30	144	48	87	170	60	68	36	163	123
8	239	37	247	235	3	110	73	206	136	81	65	107	80	219	92	229
9	100	231	50	125	24	205	96	23	230	154	72	4	57	98	146	8
10	175	152	27	249	156	28	164	55	127	177	196	116	47	216	58	124
11	115	94	169	38	108	178	148	7	253	204	54	222	203	246	217	245
12	220	157	134	242	51	106	194	45	153	78	111	18	35	118	202	114
13	0	168	238	82	31	192	59	12	180	109	208	44	221	34	49	241
14	209	135	112	104	195	67	43	76	174	225	250	11	243	69	185	29
15	16	233	210	186	56	77	6	184	120	101	84	71	79	39	248	226
16	103	138	14	240	46	66	42	88	141	200	143	90	89	13	53	132

**Table 3:** Performance measure.

Image Quality Assessment (IQA)	Suggested Method (Lena-Image)	Suggested Method (Baboon-Image)	Suggested Method (Jet-Image)	Suggested Method (Peppers-Image)
PSNR	57.80	53	56.75	54.90
SSIM	1	1	1	1

**Table 4:** Normalized correlation against image processing attacks.

Attacks	Strengths	Proposed Technique
Salt and Pepper	0.01	NC = 0.9974
Salt and Pepper	0.03	NC = 0.9950
Gaussian Noise	0.001	NC = 0.9920
Gaussian Noise	0.003	NC = 0.9915

**Table 5:** Normalized correlation against geometric attacks.

Attacks	Strengths	Proposed Technique
Cropping	25%	NC = 0.9990
Cropping	50%	NC = 0.9964
Rotation	−25	NC = 0.9980
Rotation	−50	NC = 0.9958
Translation	[25 25]	NC = 0.9980
Translation	[50 50]	NC = 0.9925

**Table 6:** Impercepability analysis.

IQA	Proposed (Lena)	Proposed (Baboon)	Baboon [26]	Baboon [34]	Baboon [25]
PSNR	57.80	53	42	53.1	49.75
SSIM	1	1	1	1	1

**Table 7:** Normalized correlation against processing attacks.

Attacks	Strengths	Proposed (Baboon)	Baboon [26]
Salt and Pepper	0.01	NC = 0.9974	NC = 0.6833
Salt and Pepper	0.03	NC = 0.9950	NC = 0.4013
Gaussian Noise	0.001	NC = 0.9920	NC = 0.9036
Gaussian Noise	0.003	NC = 0.9915	NC = 0.6974
JPEG	60%	NC = 0.9916	NC = 0.9713

**Table 8:** Normalized correlation against image processing attacks.

Attacks	Strengths	Proposed (Lena)	Lena [25]
Salt and Pepper	100%	NC = 0.9820	NC = 0.9244
Gaussian Noise	0.05	NC = 0.9910	NC = 0.9762
Gaussian Noise	0.10	NC = 0.9820	NC = 0.9561
JPEG	60%	NC = 0.9920	NC = 0.9571

**Table 9:** Normalized correlation against image processing attacks.

Attacks	Strengths	Proposed (Baboon)	Baboon [34]
Salt and Pepper	0.01	NC = 0.9974	NC = 0.9970
Salt and Pepper	0.03	NC = 0.9950	NC = 0.9948
Gaussian Noise	0.05	NC = 0.9920	NC = 0.9875
Gaussian Noise	0.10	NC = 0.9915	NC = 0.9872
JPEG	60%	NC = 0.9916	NC = 0.9916

**Table 10:** Normalized correlation against image geometrical attacks.

Attacks	Normalized Correlation			
	Proposed	Ref. [26]	Ref. [25]	Ref. [34]
Image Cropping 25%	NC = 0.9990	NC = 0.2827	-	NC = 9975
Image Cropping 50%	NC = 0.9964	-	NC = 0.9768	-
Median Filter 3 × 3	NC = 0.9960	NC = 0.3124	-	NC = 9962
Median Filter 9 × 9	NC = 0.9840	-	NC = 9130	-

## Data Availability

Not applicable.

## References

[1] Li Y. Research and application of deep learning in image recognition. Proceedings of the 2022 IEEE 2nd International Conference on Power, Electronics and Computer Applications (ICPECA).

[2] Ravindran A., George A. An Edge Datastore Architecture for {Latency-Critical} Distributed Machine Vision Applications. Proceedings of the USENIX Workshop on Hot Topics in Edge Computing (HotEdge 18).

[3] George A., Ravindran A., Mendieta M., Tabkhi H. (2021). Mez: An adaptive messaging system for latency-sensitive multi-camera machine vision at the iot edge. IEEE Access.

[4] Cao K., Liu Y., Meng G., Sun Q. (2020). An overview on edge computing research. IEEE Access.

[5] George A., Ravindran A. Distributed middleware for edge vision systems. Proceedings of the 2019 IEEE 16th International Conference on Smart Cities: Improving Quality of Life Using ICT & IoT and AI (HONET-ICT).

[6] Lenz R., Reichert M. (2007). IT support for healthcare processes–premises, challenges, perspectives. Data Knowl. Eng..

[7] George A., Ravindran A., Mendieta M., Tabkhi H. (2020). Mez: A Messaging System for Latency-Sensitive Multi-Camera Machine Vision at the IoT Edge. arXiv.

[8] Evsutin O., Melman A., Meshcheryakov R. (2020). Digital steganography and watermarking for digital images: A review of current research directions. IEEE Access.

[9] Diffie W., Hellman M.E. (2022). New directions in cryptography. Democratizing Cryptography: The Work of Whitfield Diffie and Martin Hellman.

[10] Pachghare V. (2019). Cryptography and Information Security.

[11] Bin Faheem Z., Ali A., Khan M.A., Ul-Haq M.E., Ahmad W. (2020). Highly dispersive substitution box (S-box) design using chaos. ETRI J..

[12] Farah M., Guesmi R., Kachouri A., Samet M. (2020). A new design of cryptosystem based on S-box and chaotic permutation. Multimed. Tools Appl..

[13] Lu Q., Zhu C., Wang G. (2019). A novel S-box design algorithm based on a new compound chaotic system. Entropy.

[14] Dimitrov M.M. (2020). On the design of chaos-based S-boxes. IEEE Access.

[15] Tanyildizi E., Özkaynak F. (2019). A new chaotic S-box generation method using parameter optimization of one dimensional chaotic maps. IEEE Access.

[16] Begum M., Uddin M.S. (2020). Digital image watermarking techniques: A review. Information.

[17] Garg P., Kishore R.R. (2020). Performance comparison of various watermarking techniques. Multimed. Tools Appl..

[18] Khan M.A., Khan U.A., Ali A., Hussain F., Nisar W. (2019). A robust color image watermarking scheme using chaos for copyright protection. Mehran Univ. Res. J. Eng. Technol..

[19] Luo Y., Wang F., Xu S., Zhang S., Li L., Su M., Liu J. (2022). CONCEAL: A robust dual-color image watermarking scheme. Expert Syst. Appl..

[20] Sinhal R., Jain D.K., Ansari I.A. (2021). Machine learning based blind color image watermarking scheme for copyright protection. Pattern Recognit. Lett..

[21] Li D., Deng L., Gupta B.B., Wang H., Choi C. (2019). A novel CNN based security guaranteed image watermarking generation scenario for smart city applications. Inf. Sci..

[22] Zainol Z., Teh J.S., Alawida M. (2022). An FPP-resistant SVD-based image watermarking scheme based on chaotic control. Alex. Eng. J..

[23] Hasan N., Islam M.S., Chen W., Kabir M.A., Al-Ahmadi S. (2021). Encryption Based Image Watermarking Algorithm in 2DWT-DCT Domains. Sensors.

[24] Liu Y., Tang S., Liu R., Zhang L., Ma Z. (2018). Secure and robust digital image watermarking scheme using logistic and RSA encryption. Expert Syst. Appl..

[25] Savakar D.G., Ghuli A. (2019). Robust invisible digital image watermarking using hybrid scheme. Arab. J. Sci. Eng..

[26] Singh D., Singh S.K. (2017). DWT-SVD and DCT based robust and blind watermarking scheme for copyright protection. Multimed. Tools Appl..

[27] Mokhnache S., Bekkouche T., Chikouche D. (2018). A robust watermarking scheme based on DWT and DCT using image gradient. Int. J. Appl. Eng. Res..

[28] Wang J., Du Z. (2019). A method of processing color image watermarking based on the Haar wavelet. J. Vis. Commun. Image Represent..

[29] Abraham J., Paul V. (2019). An imperceptible spatial domain color image watermarking scheme. J. King Saud-Univ.-Comput. Inf. Sci..

[30] Muyco S.D., Hernandez A.A. Least significant bit hash algorithm for digital image watermarking authentication. Proceedings of the 2019 5th International Conference on Computing and Artificial Intelligence.

[31] Singh S.P., Bhatnagar G. (2018). A new robust watermarking system in integer DCT domain. J. Vis. Commun. Image Represent..

[32] Yang S., Tan J., Chen B. (2022). Robust spike-based continual meta-learning improved by restricted minimum error entropy criterion. Entropy.

[33] Yang S., Linares-Barranco B., Chen B. (2022). Heterogeneous Ensemble-Based Spike-Driven Few-Shot Online Learning. Front. Neurosci..

[34] Hannoun K., Hamiche H., Lahdir M., Laghrouche M., Kassim S. (2018). A novel DWT domain watermarking scheme based on a discrete-time chaotic system. IFAC-PapersOnLine.

[35] Faheem Z.B., Ali M., Raza M.A., Arslan F., Ali J., Masud M., Shorfuzzaman M. (2022). Image Watermarking Scheme Using LSB and Image Gradient. Appl. Sci..

[36] Owotogbe J., Ibiyemi T., Adu B. (2019). Edge detection techniques on digital images-a review. Int. J. Innov. Sci. Res. Technol..

[37] Kumar M., Kumar R., Yadav J. (2020). A robust digital speech watermarking based on least significant bit. Int. J. Innov. Technol. Explor. Eng. (IJITEE).

[38] Singh R.K., Dube A.P., Singh R. (2022). Least Significant Bit-Based Image Watermarking Mechanism: A Review. Int. J. Soc. Ecol. Sustain. Dev. (IJSESD).

[39] Shannon C.E. (1949). Communication theory of secrecy systems. Bell Syst. Tech. J..

[40] Özkaynak F. (2020). On the effect of chaotic system in performance characteristics of chaos based s-box designs. Phys. A Stat. Mech. Its Appl..

